# Development of a Point-of-Care Cervico-Vaginal Sampling/Testing Device for the Colorimetric Detection of Cervical Cancer

**DOI:** 10.3390/diagnostics13081382

**Published:** 2023-04-10

**Authors:** Tejaswini Appidi, Murali Vakada, Hima Sree Buddhiraju, Shubham A. Chinchulkar, Akshar Kota, Dokkari Nagalaxmi Yadav, Suseela Kodandapani, Surya Kumar Simhabhatla, Aravind Kumar Rengan

**Affiliations:** 1 Department of Biomedical Engineering, Indian Institute of Technology Hyderabad, Kandi 502284, India; 2 Department of Mechanical and Aerospace Engineering, Indian Institute of Technology Hyderabad, Kandi 502284, India; 3 Department of Pathology, Basavatarakam Indo-American Cancer Hospital & Research Institute, Hyderabad 500034, India

**Keywords:** point-of-care device, cervico-vaginal fluid screening, self-sampling device, gold nanoparticles, colorimetric detection, cervical cancer

## Abstract

This paper reports the **col**orimetric analysis of **c**ervical-cancer-affected clinical samples by the in situ formation of gold nanoparticles (**Au**NPs) formed with cervico-vaginal fluids collected from healthy and cancer-affected patients in a clinical setup, termed “**C**-Col**Au**r”. We evaluated the efficacy of the colorimetric technique against the clinical analysis (biopsy/Pap smear) and reported the sensitivity and specificity. We investigated if the aggregation coefficient and size of the nanoparticles responsible for the change in color of the AuNPs (formed with clinical samples) could also be used as a measure of detecting malignancy. We estimated the protein and lipid concentrations in the clinical samples and attempted to investigate if either of these components was solely responsible for the color change, enabling their colorimetric detection. We also propose a self-sampling device, **C**ervi**S**elf, that could enable the rapid frequency of screening. We discuss two of the designs in detail and demonstrate the 3D-printed prototypes. These devices, in conjugation with the colorimetric technique **C**-Col**Au**r, have the potential to be self-screening techniques, enabling women to undergo rapid and frequent screening in the comfort and privacy of their homes, allowing a chance at an early diagnosis and improved survival rates.

## 1. Introduction

Cervical cancer is the fourth most common cancer and the fourth leading cause of cancer death among women, with 604,127 new cases and 341,832 deaths recorded globally in 2020 (GLOBOCAN 2020) [1,2,3,4]. It is the second most common cancer among Indian women, and the burden of cervical cancer is exceptionally high in low- and middle-income countries, owing to a lack of awareness, socio-economic and cultural barriers leading to delayed screening. Cervical cancer is largely caused by the human papillomavirus (HPV) [5,6]. Organized screening and vaccination programs were reported to reduce mortality and incidence in developed nations [7]. Cervical cytologic testing, including the Papanicolaou (Pap or Pap smear) test, visual inspection with acetic acid (VIA), biopsy, and primary HPV gene testing are the classic screening and diagnostic methods adopted by developed nations, which helped decrease the prevalence of cancer over time [8,9,10,11]. The same could not be implemented in low- and middle-income countries due to the expenses involved and the resources required [12]. There have been numerous reports of developing affordable screening techniques for resource-limited settings, along with various studies reporting the use of computer-based intelligence and machine learning to develop automated screening methods that can assist detecting cervical cancer [9,10,13]. However, these screening techniques would require more research to be integrated successfully into healthcare. The limitations of current screening tests and their disadvantages, including discomfort, pain, and inconvenience, also limit women from undergoing screening. Hence, there is an immediate need for a rapid point-of-care testing method that is both accessible and affordable. Earlier, we reported one such technique, “**C**-**C**ol**Au**r”, which is a simple colorimetric technique that demonstrated an appreciable sensitivity and specificity for both the diagnosis and prognosis using clinical samples. With a simple and more accessible approach and without a need for pre-processing of samples or post-processing for interpretation of results, **C**-**C**ol**Au**r shows a visible difference between cancer-affected and healthy cervico-vaginal samples within two minutes [14].

Cervical cancer self-sampling kits are also a revolutionary new way to test for cervical cancer in the privacy of one’s home. Self-sampling kits are a simple, non-invasive way for women to test for the presence of human papillomavirus (HPV), which is the leading cause of cervical cancer [15,16]. There are a few commercially available cervical cancer self-sampling kits [17,18]. The use of self-sampling kits is especially helpful in increasing access to cervical cancer screening for women who are unable or unwilling to access medical personnel for a Pap test. A survey showed that when offered a choice of HPV self-sampling or traditional screening with a Pap smear, two-thirds of participants selected HPV self-sampling, suggesting that self-sampling may be a more acceptable and convenient option, indicating the acceptability and feasibility of self-sampling [19]. Self-sampling could also be a cost-efficient approach as compared with the conventional screening techniques [20]. All the available self-sampling techniques allow the woman to take a sample herself, in the privacy and comfort of her home or office, at her convenience, and mail it to a facility which then performs the HPV testing and mails the results [21]. The design of cervical cancer self-sampling kits is constantly evolving as researchers continue to develop new and improved methods. While all the self-sampling kits have the same goal, to collect a sample of cervical cells for examination, there has been no way of analyzing these samples instantaneously to date. In all the cases, the collected samples are transported to another facility for their testing. In this context, a simple approach to analyze the collected samples using a colorimetric technique for a preliminary screening could encourage women to undergo the screening more frequently. This would further revolutionize the screening process, encouraging and improving the frequency of testing, allowing for an early diagnosis. A technique such as this could be more useful in resource-limited conditions. The affordability of self-sampling kits is also an important factor in their effectiveness. In this paper, we propose and introduce a self-sampling device, **C**ervi**S**elf, for the **self**-sampling of **cervi**co-vaginal fluids.

In this paper, we report the colorimetric analysis of cervico-vaginal fluids collected from healthy and cancer-affected patients in a clinical setup using the **C**-Col**Au**r technique. We evaluated the cumulative specificity and sensitivity using all the collected clinical samples to date. We also investigated if the aggregation coefficient and size of the nanoparticles (formed with clinical samples) could also be used as a measure of detecting malignancy. We estimated the protein and lipid concentrations in the clinical samples and attempted to investigate if either of these intracellular components was solely responsible for the color change, enabling their colorimetric detection. In addition, we also propose a self-sampling device that could allow the rapid frequency of screening in conjugation with the colorimetric technique. We discuss two of the designs in detail and demonstrate the 3D-printed prototypes. In conjugation with the colorimetric method **C**-Col**Au**r, these devices have the potential to be self-screening techniques, enabling women to undergo rapid and frequent screening in the comfort and privacy of their homes, allowing a chance at an early diagnosis and improved survival rates.

## 2. Materials and Methods

### 2.1. Materials

Tetrachloroauric acid trihydrate (HAuCl_4_·3H_2_O), Bovine serum albumin, and ascorbic acid were purchased from Sigma Aldrich Pvt. Ltd., St. Louis, MI, USA. Phospholipid quantification assay kit (CS0001-1KT) estimation kits were purchased from Sigma (St. Louis, MO, USA). A BCA protein assay kit was purchased from Thermofisher Ltd., Hyderabad, India. The synthetic lipid L-α-phosphatidylcholine, hydrogenated (Soybean) HSPC, was a generous gift from Lipoid, Germany. All the chemicals were used as received without any further purification.

### 2.2. Characterization

The clinical samples were characterized for their UV absorbance using a UV−vis spectrophotometer (Shimadzu UV-1800, Shimadzu Corporation, Kyoto, Japan). The clinical samples’ hydrodynamic diameter and polydispersity index (P.D.I.) were evaluated using the dynamic light scattering technique (Particle Sizing Systems, Inc., Santa Barbara, CA, USA). The morphology and size of clinical samples and the nanoparticles formed with clinical samples were visualized using a scanning electron microscope, FESEM (Carl Zeiss atomic microscope, Supra40, Carl Zeiss AG, Germany), and a transmission electron microscope (TEM) (JEOL, J.E.M. 2100F Inc., Peabody, MA, USA).

### 2.3. Clinical Samples: Collection and Analysis

The clinical samples were obtained from the Basavatarakam Indo-American Cancer Hospital and Research Institute in Hyderabad, Telangana, India, after acquiring all the necessary ethical permissions. Written informed consent was acquired (registration number: ECR/7/Inst/AP/2013/RR-16) from each patient. The gynecologists collected the cervico-vaginal swabs using a cytobrush, with patients in the lithotomy posture. Additionally, appropriate samples for Pap smears or biopsies for clinical analysis were also collected. The samples collected using the cytobrush were carefully transferred to sterile tubes containing autoclaved Milli Q water. The tubes were sealed, labelled, and transported to the lab in a storage box containing ice packs and stored at −80 °C for further analysis. A total of 20 samples were obtained and reported in this study. The Pathology lab, Department of Molecular Diagnostics, Basavatarakam Indo-American Cancer Hospital and Research Institute, Hyderabad, analyzed all clinical cytology samples collected from patients for Pap smears/biopsies for screening and diagnosis. Healthy and cervical-cancer-affected women were identified based on the clinical analysis (Pap smear/biopsy results), and the findings with **C**-Col**Au**r were compared [14].

### 2.4. Analysis of Clinical Samples Using the **C**-Col**Au**r Technique

The cervical fluid samples were diluted in autoclaved Milli Q water (1:10). As reported in our earlier publication, the clinical samples were analyzed for color change using colorimetric detection via the formation of in situ gold nanoparticles (AuNPs) [14]. Briefly, 100 µL of the diluted clinical sample was added to 100 µL of 1 mM of HAuCl_4_·3H_2_0, followed by 200 µL of 2 mM ascorbic acid. A rapid change in color with the addition of ascorbic acid was observed. For ease of comparison and incorporation of earlier found results, we used the same terminology for the various nanoparticles. The AuNPs obtained without any clinical samples were referred to as blank (B) NPs, and the AuNPs obtained with healthy and cancer-affected cervical fluids were termed as control (C) and test (T) AuNPs, respectively.

### 2.5. Sensitivity and Specificity of **C**-Col**Au**r Detection

The **C**-Col**Au**r technique’s sensitivity, specificity, negative predictive value (NPV), and positive predictive value (PPV) were determined using the standard methods described in the literature [22,23]. The Pap smear and biopsy results were considered the gold standard. We analyzed a total of 20 new samples in this report. We also added the earlier reported samples (*n* = 62) to the current samples (*n* = 20) and calculated a cumulative specificity and sensitivity for a total of 82 samples [14]. The calculations are shown in the ESI.

### 2.6. Aggregation Coefficient and Particle Size Analysis

In addition to the colorimetric detection, we attempted to evaluate and understand if the aggregation coefficient (a relative absorbance parameter calculated from UV-visible spectroscopy) and particle size of the nanoparticles formed with clinical samples could also be used as a measure of detection [24,25]. We analyzed a total of 42 samples, out of which 12 were healthy samples and 30 were affected samples. For the calculation of the aggregation coefficient (A_520_/A_580_), the absorbance at 520 nm and 580 nm was recorded for all the AuNPs formed with (healthy and affected) and without clinical samples. The absorbance at 520 nm corresponded to the λmax of blank AuNPs (AuNPs formed without any clinical sample), while the absorbance at 580 nm corresponded to the λmax of test nanoparticles (AuNPS formed with cervical-cancer-affected clinical samples). The ratios thus calculated were plotted as mean ± SEM and analyzed for statistical significance.

### 2.7. Estimation of the Concentration of Lipids and Protein in Clinical Samples

The protein and lipid contents in the clinical samples were estimated. The diluted clinical samples were subjected to the BCA protein and phospholipid assays following the manufacturer’s instructions [14]. A total of 42 samples were analyzed, out of which 12 were healthy samples and 30 were affected samples. The concentrations of lipid and protein were plotted for healthy and affected samples. The average concentration of lipids and proteins was also calculated for further experiments with synthetic proteins and lipids.

### 2.8. Analysis with Synthetic Proteins and Lipids

To understand if proteins or lipids in the clinical samples were solely responsible for the difference in color achieved with the type of sample (i.e., blue color for a healthy sample and colorless for a cervical-cancer-affected sample), we analyzed the collected clinical samples. Following the results from the estimated lipid and protein concentrations in clinical samples, we attempted to perform the **C**-Col**Au**r technique using synthetically available lipids and proteins at the same concentrations (average concentrations of proteins and lipids of the healthy and affected samples). The average concentrations of protein in healthy (*n* = 12) and affected (*n* = 30) cervico-vaginal fluid samples were calculated as 466.2 ± 128.5 mg/mL and 1218 ± 125 mg/mL, respectively. The average concentrations of lipids in healthy (*n* = 12) and affected (*n* = 30) cervico-vaginal fluid samples were found to be 2.76 ± 1.15 µM/µL and 12.41 ± 1.57 µM/µL. We used the synthetic protein and lipids albumin and HSPC, respectively, for the analysis. Briefly, the average concentrations of lipids and proteins were prepared, and the same procedure was followed as for **C**-Col**Au**r, except substituting the clinical sample for either a synthetic lipid or protein or both. In addition to the only lipid and only protein, we also mixed the lipid and the protein in a 1:1 ratio and followed the same procedure to observe the color change, if any. The color change was recorded along with the absorbance and size of the particles.

### 2.9. Design of the Self-Sampling Device: CerviSelf

#### 2.9.1. Design Specifications

The designs presented in this work are devices to collect endocervical cells from the target region for the early detection of cervical cancer. The current designs were generated considering a two-step approach: concept generation and critical evaluation. Various possible design ideas that met the research objectives were proposed and compared. The generated designs were scrutinized by analyzing the project’s functional requirements and the users’ ease of use, comfort, privacy, and affordability [26]. Two alternative designs were ultimately chosen for evaluation from a pool of six different designs based on the requirements. The modeling of the design ideas was carried out in a mechanical CAD environment using the Solidedge software. A set of specifications and requirements for the design and development of this device was established based on the state-of-the-art literature review and clinical expertise [27]. The following design specifications were identified and evaluated: functionality, comfort, convenience, affordability, reliability, and sample collection without contamination.

#### 2.9.2. Physical Prototypes

The physical prototypes of the selected designs were 3D printed using an HP Jet Fusion 580, a multi-Color Binder Jetting 3D Printer at IIT Hyderabad, Telangana, India.

### 2.10. Statistical Analysis

Mean ± SEM values were used for the expression of the data. The statistical analyses of the data were performed using the Graphpad prism, and values of *p* < 0.05 were considered statistically significant.

## 3. Results and Discussions

### 3.1. Clinical Samples and Their Analysis

**Figure 1:**
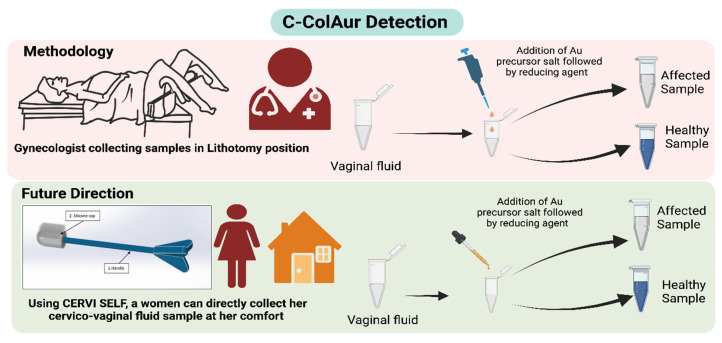
Schematic explaining the **C**-Col**Au**r detection and its future direction using **C**ervi**S**elf.

The typical approach of colorimetric assays involves the preparation of a stable colloidal solution/substrate, whose stability is intervened with by the addition of the analyte resulting in a color change. The majority of the reported studies on colorimetric analysis for biosensing use pre-formed AuNPs [24,28,29,30,31], while the **C**-Col**Au**r technique is based on the in situ formation of AuNPs in the presence of clinical samples (cervico-vaginal fluids) collected from both healthy and cancer-affected subjects. **C**-Col**Au**r is a colorimetric strategy relying on the in situ formation of AuNPs that vary in size and color depending upon the type of clinical sample leading to cervical cancer screening [14]. A schematic representation of the **C**-Col**Au**r technique is shown in Figure 1. The samples were collected by the gynecologists in a lithotomy position, and these samples were evaluated by both the **C**-Col**Au**r technique and clinical analysis (conventional diagnostic techniques such as Pap smears/biopsies). As for the **C**-Col**Au**r technique, the clinical samples, when reacted with gold salt and ascorbic acid, showed an immediate color change. The healthy samples turned blue, while the affected samples remained colorless.

**Figure 2:**
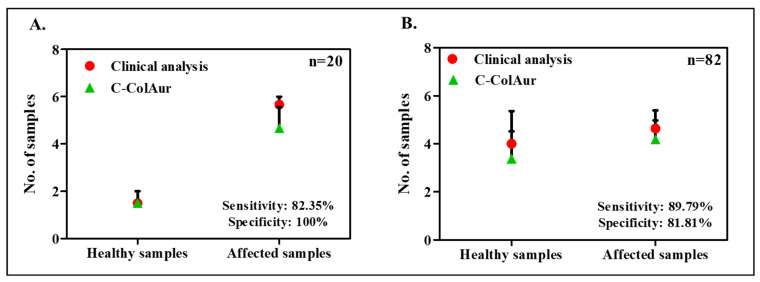
Comparison of **C**-Col**Au**r with clinical analysis for (**A**) new samples (*n* = 20) and (**B**) total samples (*n* = 82).

In this paper, we report the analysis of 20 new samples using **C**-Col**Au**r, and the results of the **C**-Col**Au**r technique were compared with the clinical analysis (of a biopsy or pap smear). The results are furnished in SI Table S1. A sensitivity of 82.35% and specificity of 100% were recorded for these samples (*n* = 20), considering a biopsy as a gold standard (SI Table S2), as shown in Figure 2A. We further calculated the cumulative sensitivity and specificity, combining the results of all the samples collected to date (current samples (*n* = 20) and earlier reported samples (*n* = 62)). The sensitivity and specificity of earlier samples (*n* = 62) as shown in SI Table S3 are calculated to be 93.75% and 80%, respectively. We report a cumulative sensitivity of and specificity for total samples (*n* = 82) as 89.79% and 81.81%, respectively (SI Table S4 and Figure 2B).

**Figure 3:**
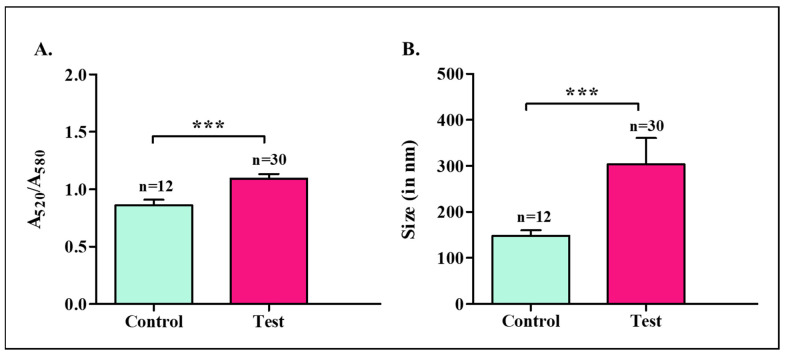
(**A**). Aggregation coefficient (ratio of spectral intensities at 520 nm to 580 nm) (*** *p* = 0.0002) and (**B**) size analysis of control and test nanoparticles (gold nanoparticles formed with healthy and cancerous samples, respectively) (*** *p* = 0.0001).

We investigated if the aggregation coefficient and size of the nanoparticles, which are responsible for the change in color of the nanoparticles formed with clinical samples, could also be used as a measure of detection, in addition to the visual colorimetric detection. The aggregation coefficient is a relative measure of absorbance, i.e., A_520_/A_580_ (λmax of blank nanoparticles/λmax of test nanoparticles), and was calculated for nanoparticles formed with clinical samples. The standard absorbance of gold nanoparticles was recorded at 520 nm, and the shift in the wavelength for healthy and affected samples was fixed at 580 nm. The blank nanoparticles showed a maximum absorbance at 520 nm, and the test nanoparticles showed a maximum absorbance at 580 nm [14]; hence, the ratio of relative absorbance at A_520_/A_580_ was used as the aggregation coefficient. The aggregation coefficient for the clinical samples was calculated from their respective UV-visible absorbance spectra. The aggregation coefficient of the samples formed with healthy cervico-vaginal fluids was 0.8625 ± 0.0460, while that of cancer-affected samples was 1.0930 ± 0.0390. A significant difference in the aggregation coefficient for the nanoparticles formed with healthy and cancer-affected samples was recorded, as shown in Figure 3A. We also evaluated the size of the control and test nanoparticles (nanoparticles formed with healthy and affected samples, respectively), and the data are shown below in Figure 3B. The average size of the nanoparticles was measured using a particle size analyzer. The mean diameter of the control nanoparticles was 148.70 ± 11.45 nm, and that of the test nanoparticles was 303.5 ± 57 nm. There was a significant difference between the sizes of the nanoparticles formed with healthy and affected samples.

This thereby suggested that these two parameters, the aggregation coefficient and size of the nanoparticles, could also be used as a measure of detection. The SEM and TEM micrographs of the AuNPs formed with healthy and affected samples are shown in SI Figures S1 and S2.

**Figure 4:**
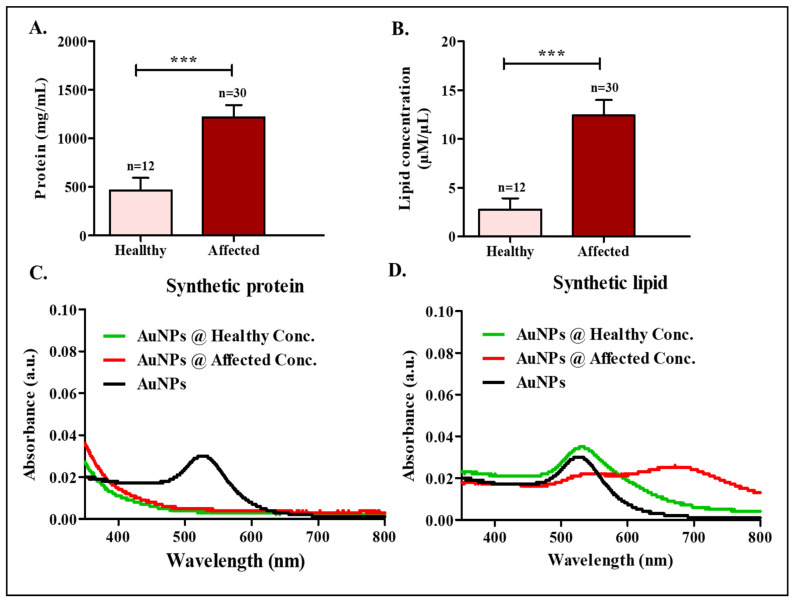
Estimation of (**A**) protein and (**B**) lipid in clinical samples (*** *p* = 0.0003 for protein analysis and *p* = 0.0001 for lipid analysis). Absorbance spectra of AuNPs formed with (**C**) synthetic protein and (**D**) synthetic lipid at average concentrations found in clinical samples.

The SEM micrographs of the clinical samples are shown in SI Figure S3. The difference in the number of cells and the concentration of the intracellular constituents such as proteins and lipids was hypothesized to be the reason for the change in the color observed in the **C**-Col**Au**r technique [14]. We estimated the protein and lipid concentrations in healthy and cancer-affected cervico-vaginal fluids. The average values of protein and lipid contents in both healthy and cancer-affected samples are shown in Figure 4A,B, respectively. The mean protein concentration in healthy samples was found to be 466.2 ± 128.5 mg/mL, while that found in cancerous samples was 1218 ± 125.3 mg/mL. The lipid was also estimated in the samples, and the average lipid concentration was found to be 2.76 ± 1.15 µM/µL for healthy samples and 12.41 ± 1.57 µM/µL for cancerous samples. There was a significant difference in the concentrations of the lipids and proteins, which played a potential role in enabling colorimetric detection by affecting the size and shape of the nanoparticles formed. We further tried to understand if the color change could be attributed solely to either proteins or lipids. We performed the following experiment to determine if the lipid and protein could independently cause the color change. We used synthetically available protein (albumin) and lipid (HSPC) in the same concentrations as the average concentrations found in the healthy and cancer-affected samples and reacted them independently with the reagents (HAuCl_4_.3H_2_0 and ascorbic acid) to understand the color change. In short, we repeated the **C**-Col**Au**r technique, with synthetic lipid or/and protein at the same average concentrations found in clinical samples. A change in color was recorded (SI Figure S4) along with their absorbance spectra and size analyses. For the analysis with protein alone, two concentrations of bovine serum albumin (BSA), i.e., 466.2 mg/mL and 1218 mg/mL, corresponding to healthy and cancer-affected samples, were chosen, respectively. Upon their reaction with gold salt and ascorbic acid, no change in color was observed (SI Figure S4A). The samples were further analyzed for their absorbance spectra and size (Figure 4C and SI Table S6). The color change and absorbance spectra did not correlate with the results observed with the clinical samples [14], leading to the inference that the color change could not just be attributed to the protein concentration in the clinical samples.

We performed a similar experiment with synthetic lipid (HSPC). Two concentrations of HSPC lipid, i.e., 2.76 mM/mL and 12.4 mM/mL, corresponding to the average concentrations of healthy and cancer-affected samples, were chosen. The change in color upon reaction with gold salt and ascorbic acid was recorded, and the samples were further analyzed for their absorbance spectra and size (SI Figure S4 and Table S6). A difference in color and a difference in the absorbance spectra (Figure 4D) were observed for the two concentrations of lipid used but did not precisely correlate with the results observed with the clinical samples, suggesting that the change in color could also not be attributed to the concentration of the lipids only. In addition to only lipids and only proteins, we also evaluated if a mixture of lipids and proteins in the ratio of 1:1 could yield the same results as **C**-Col**Au**r. Upon reaction with gold salt and ascorbic acid, there was no change in color observed for these mixtures. The UV absorbance spectra also did not show any significant differences (SI Figure S5). From these above experiments, we understood that either the lipid or protein content alone in the clinical samples could not be the sole reason for the difference in color-enabling detection. A combination of proteins and lipids could be responsible for the change in color along with various other biological components in the cervico-vaginal fluids, playing an essential role in enabling the detection.

### 3.2. Modeling, Design, and Prototyping of a Self-Sampling Device

**Figure 5:**
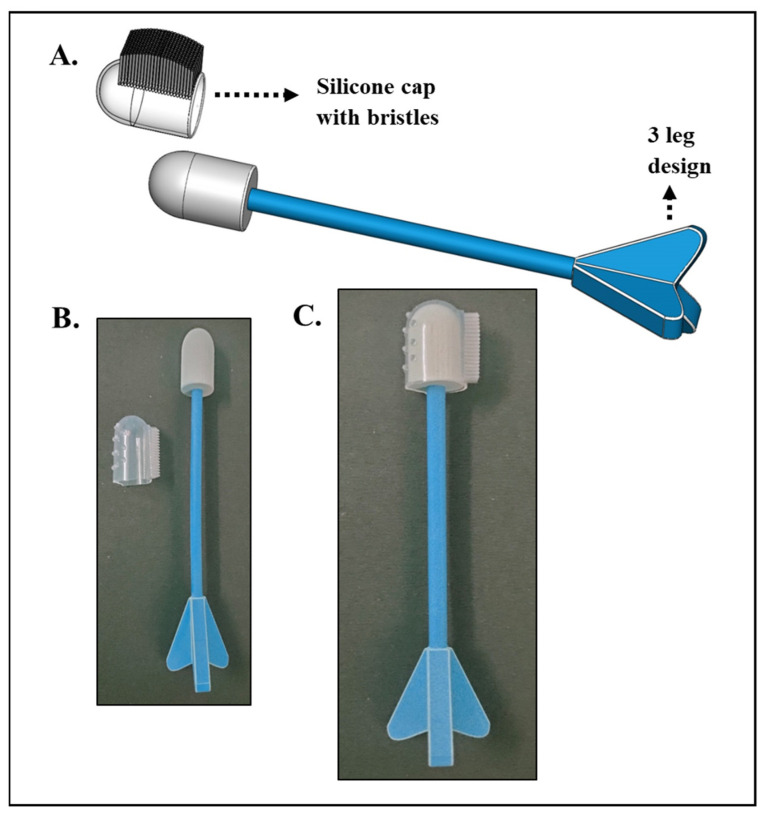
**C**ervi**S**elf design 1. (**A**) Design. (**B**,**C**) Prototypes of the design with silicone bristles.

Self-sampling kits have revolutionized cervical cancer screening, by offering comfort, convenience, and privacy, allowing women to collect their own samples. Self-sampling kits aim to collect the samples, and how they achieve this goal varies. Some kits use a brush to collect cells from the cervix, while others use a swab. The brush or swab should be able to collect cells from the cervix without causing pain or discomfort. The kit should also be easy to use, with clear instructions on how to collect the sample and send it for examination. Self-sampling kits are also a more affordable option as they can be purchased for a fraction of the cost of a traditional screening [32]. In this paper, we report a novel design for a self-sampling device, **C**ervi**S**elf, a device for self-sampling of cervico-vaginal samples, and demonstrate 3D printed prototypes. These devices were designed and developed such that the samples could be collected and transported or analyzed with the **C**-Col**Au**r technique. We modeled, designed, and prototyped self-sampling devices. The kits were designed to be user-friendly and easy to use, with an aim to make women feel comfortable and confident while using them. The current designs were generated considering a two-step approach: concept generation and critical evaluation. Various possible design ideas that met the research objectives were proposed and compared. The generated designs were scrutinized by analyzing the project’s functional requirements and the users’ ease of use, comfort, privacy, and affordability [26]. Various iterations were performed, and weighing on the design and manufacturing for ease, comfort, and affordability, two designs that could be commercialized for self-sampling are discussed here. The various designs developed are given in SI Table S7. Two alternative designs were ultimately chosen for evaluation from a pool of six different designs based on the requirements. Figure 5 depicts an overview of one of the design ideas. It is a two-component device: (1) a handle with three legs design, which offers a firm grip to the user to control the movements of the brush from entry to up to the target region, and (2) a silicone bristle cap with soft bristles on top, which helps scratch the target area gently to collect the sample cells. The flexible silicone cap fits perfectly to the handle, giving users a more convenient usage than any other collection device on the market. The current design offers a 360° rotation for maximum recovery of endocervical cells. In addition, it is a cost-effective device with silicone caps that can be separated easily from the handle after sample collection without contamination. A user can use this device in four practical steps: (1) attaching the silicone cap onto the handle, (2) insertion, (3) withdrawal after sample collection, and (4) separating the cap from the handle.

**Figure 6:**
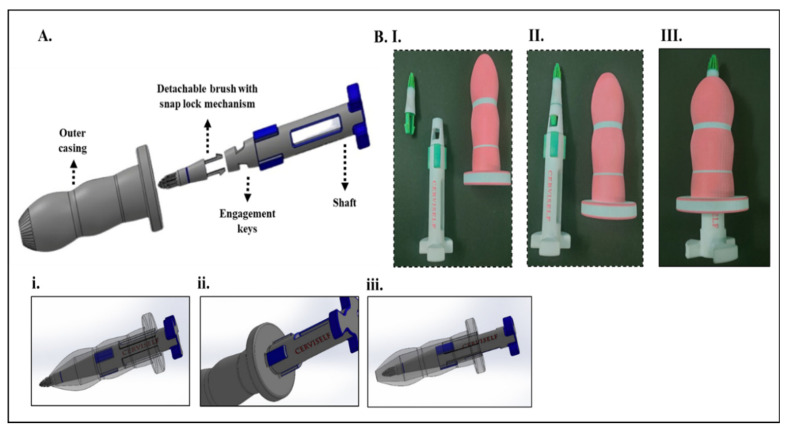
CerviSelf design 2. (**A**) CAD model showing different components. Figures showing (**i**) locking and insertion, (**ii**) alignment of keys, and (**iii**) withdrawal of brush. (**B**) 3D printed prototype of the design showing (**I**) individual components, (**II**) alignment of head(with bristles) and handle, and (**III**) fully fixed design ready for insertion.

Figure 6 shows the assembly view of another design idea that consists of three components: (1) an outer covering, with a user-friendly shape; (2) a rotating shaft; and (3) a detachable brush that can be detached easily. The rotating shaft has engagement keys on the body that align with the slots inside the outer covering. After inserting a certain length, the sample collector can be rotated and moved forward by the controlled movements of the hand. The main advantage of this design is having a detachable brush connected to the shaft, which can be removed after sample collection without contamination. A simple snap lock mechanism is used in this design to separate the brush from the shaft. First, the outer casing is inserted inside, which holds the vaginal walls in position, and then a simple five-step procedure is followed for the sample collection: (1) locking the detachable brush onto the shaft, (2) inserting the shaft into the outer case by aligning the engagement keys, (3) rotating the shaft clockwise or counterclockwise for adequate sample collection, (4) withdrawing the shaft, and (5) separating the brush from the shaft. The detailed dimensions and the materials used for prototyping the devices are given in SI Figures S6 and S7. In addition, a comparison of the **C**ervi**S**elf designs with the existing sample collecting units used in clinics are given in SI Table S8.

We propose to use the above discussed self-sampling devices in conjugation with our colorimetric technique, providing an opportunity for women to evaluate their samples instantaneously. Our design process also ensures and provides for sample transportation to a facility for analysis. The **C**ervi**S**elf, in combination with **C**-Col**Au**r, could revolutionize cervical cancer screening. The collection process is simple, and colorimetric analysis could encourage women to undergo rapid screening and pose an opportunity for the early diagnosis of the disease. This could be very helpful in resource-limited conditions, where a simple analysis could show the presence of malignancy. The **C**-Col**Au**r technique, with an appreciable sensitivity and specificity, could be an affordable solution for low- and middle-income countries to address the problem and burden of cervical cancer.

## 4. Conclusions

In this paper, we reported the colorimetric analysis of clinical samples for cervical cancer screening and showed a specificity of 81.81% and sensitivity of 89.79% for a total of 82 samples. We also demonstrated that the two important parameters, the aggregation coefficient (a measure of relative absorbance of blank and test nanoparticles) and size of the nanoparticles formed with clinical samples, showed a significant difference, validating their colorimetric difference. The color difference between the gold nanoparticles formed with healthy (blue) and affected (colorless) samples was due to the size of the nanoparticles, causing a shift in their wavelength, which was validated by the size analysis and aggregation coefficient, respectively. A considerable difference between these two parameters for 42 samples further validated and quantified the detection technique. We also estimated the protein and lipid content in the clinical samples, to show the differences among the healthy and cancer-affected samples. We also attempted to understand if the protein or lipid content of the clinical samples was solely responsible for the color change observed using synthetic proteins and lipids. In addition to these, we also designed and developed a self-sampling device, **C**ervi**S**elf, and we propose that this device, in combination with the colorimetric technique **C**-Col**Au**r, could revolutionize cervical cancer screening in low- and middle-income countries.

## Data Availability

Not applicable.

## Supplementary Materials

The following supporting information can be downloaded at: https://www.mdpi.com/article/10.3390/diagnostics13081382/s1. The clinical sample analysis, calculation of sensitivity and specificity, SEM and TEM micrographs of clinical samples and AuNPs formed with clinical samples, Images showing the change in color with synthetic lipids and proteins and their characterization is given [14].
