# Supplementary material for: Development of a Point-of-Care Cervico-Vaginal Sampling/Testing Device for the Colorimetric Detection of Cervical Cancer
Source: Diagnostics (Basel). 2023 Apr 10;13(8):1382. doi: 10.3390/diagnostics13081382 (PMC10137237; doi:10.3390/diagnostics13081382)
Supplement: Supplementary file 1 [file diagnostics-13-01382-s001.zip › diagnostics-2282400-supplementary.pdf]

Development of a point-of-care cervico-vaginal sampling/testing device for the colorimetric detection of cervical cancer

Tejaswini Appidi[a#], Murali Vakada[b#], Himasree Buddhiraju[a#], Shubham A Chinchulkar[a], Akshar Kota[b], Nagalaxmi Yadav Dokkari[a], Suseela Kodandapani[c], Surya Kumar Simhabhatla[b]*, Aravind Kumar Rengan[a]*

a. Dept. of Biomedical Engineering, Indian Institute of Technology Hyderabad, India.

b. Dept. of Mechanical and Aerospace Engineering, Indian Institute of Technology Hyderabad, India

c. Dept. of Pathology, Basavatarakam Indo-American Cancer Hospital & Research Institute, Hyderabad, India.

*Corresponding author email: aravind@bme.iith.ac.in

Co-Corresponding author email: ssurya@mae.iith.ac.in

[#]Authors have contributed equally.

Supporting Information

SI Table S1: **Comparison of "C-ColAur" results against clinical analysis (Biopsy/ Pap smear) for the collected clinical samples (new samples n=20).**

S. No	Date of sample collection	Sample ID	Clinical report	C-ColAur technique
1.	8/9/2022	08/02/MR47	+Ve	+Ve
2.	8/9/2022	08/03/MR52	+Ve	+Ve
3.	8/3/2022	08/01/MR38	-Ve	-Ve
4.	8/30/2022	08/05/MR80	+Ve	+Ve
5.	8/30/2022	08/06/MR68	+Ve	+Ve
6.	8/30/2022	08/07/MR61	+Ve	+Ve
7.	8/25/2022	08/04/MR77	+Ve	+Ve
8.	9/13/2022	09/01/MR19	+Ve	+Ve
9.	9/13/2022	09/02/MR24	+Ve	+Ve
10.	9/20/2022	09/04/MR92	_-Ve	-Ve
11.	9/20/2022	09/05/MR16	+Ve	-Ve
12.	9/20/2022	09/06/MR26	+Ve	+Ve
13.	9/20/2022	09/07/MR14	+Ve	-Ve
14.	9/20/2022	09/08/MR47	-Ve	-Ve
15.	9/20/2022	09/09/MR11	+Ve	-Ve
16.	10/18/2022	10/01/MR92	+Ve	+Ve
17.	10/18/2022	10/02/MR92	+Ve	+Ve
18.	10/18/2022	10/03/MR43	+Ve	+Ve
19.	10/18/2022	10/04/MR89	+Ve	+Ve
20.	10/18/2022	10/06/MR22	+Ve	+Ve

*+Ve: Positive and –Ve: Negative.

SI Table S2: **Calculation of sensitivity and specificity for new samples (n=20).**

	**Gold standard (Disease present)**	**Gold standard (Disease absent)**	
Test Positive	True Positives (a): 14	False positives (b): 0	Total test positives(a+b): 14
Test Negative	False negatives (c):3	True negatives (d): 3	Total test negatives(c+d): 6
	Total diseased (a+c): 17	Total normal (b+d): 3	Total population (a+b+c+d): 20

Sensitivity: a/(a+c): **82.35%**

Specificity: d/(b+d): **100%**

Positive Predictive Value: a/(a+b): **100%**

Negative predictive value: d/(c+d): **50%**

SI Table S3: **Calculation of sensitivity and specificity for earlier samples (n=62) [1].**

	**Gold standard (Disease present)**	**Gold standard (Disease absent)**	
Test Positive	True Positives (a): 27+3=30	False positives (b): 4+2=6	Total test positives(a+b): 31+5=36
Test Negative	False negatives (c):1+1=2	True negatives (d): 10+14=24	Total test negatives(c+d): 11+16=26
	Total diseased (a+c): 28+4=32	Total normal (b+d): 14+16=30	Total population (a+b+c+d): 62

*The non-highlighted values correspond to pre-treatment samples, while the highlighted values in yellow correspond to post-treatment samples

Sensitivity: a/(a+c): 30/32= **93.75%**

Specificity: d/(b+d): 24/30= **80%**

Positive Predictive Value: a/(a+b): **83.33 %**

Negative predictive value: d/(c+d): **92.30%**

SI Table S4: **Calculation of sensitivity and specificity for all samples (n=82).**

	**Gold standard (Disease present)**	**Gold standard (Disease absent)**	
Test Positive	True Positives (a): 30+14 =44	False positives (b): 6+0 =6	Total test positives(a+b): 36+14 = 50
Test Negative	False negatives (c):2+3=5	True negatives (d): 24+3 =27	Total test negatives(c+d): 26+6 = 32
	Total diseased (a+c): 32+17=49	Total normal (b+d): 30+3=33	Total population (a+b+c+d): 50+32 = 82

*The highlighted values are the values corresponding to new samples (n=20; from Table 2).

Sensitivity: a/(a+c): = **89.79%**

Specificity: d/(b+d): **81.81%**

Positive Predictive Value: a/(a+b): **88 %**

Negative predictive value: d/(c+d): **84.37%**

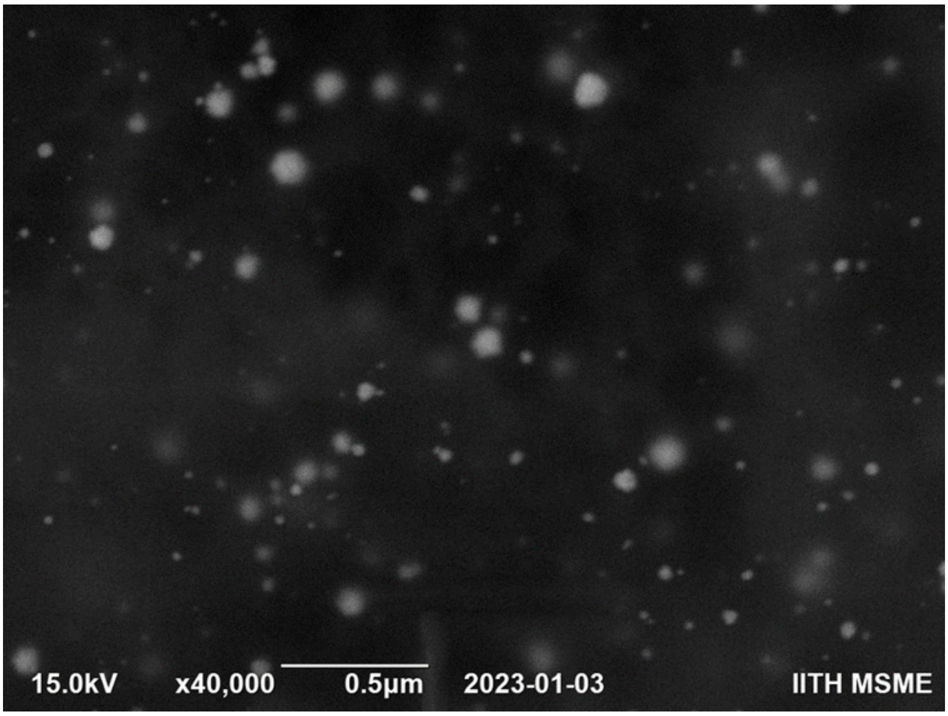

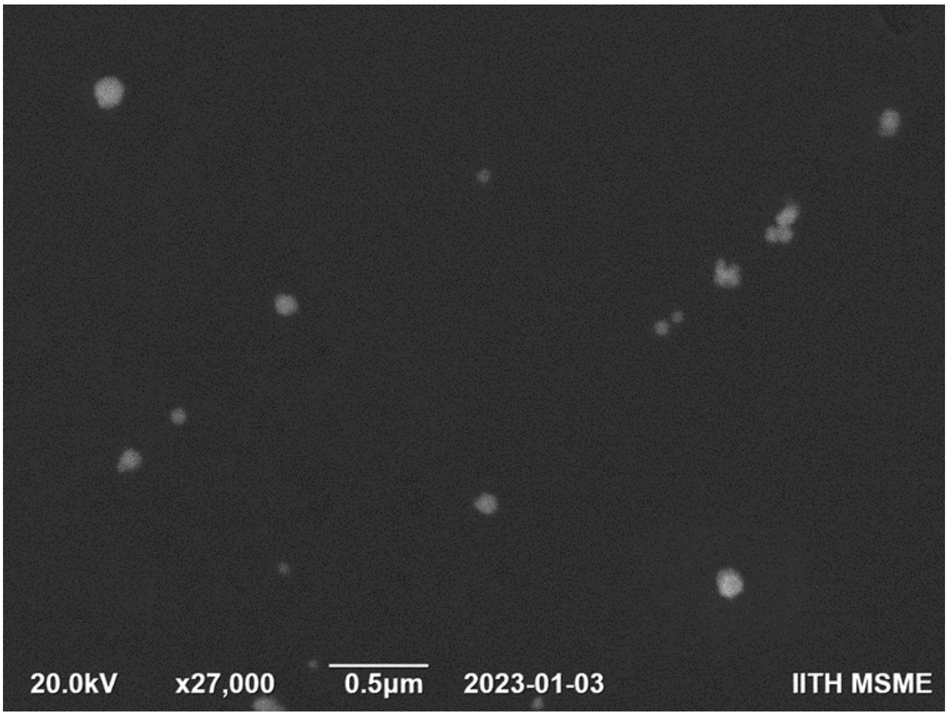

SI Figure S1: The SEM micrographs of AuNPs formed with healthy (top) and cervical cancer affected (bottom) clinical samples.

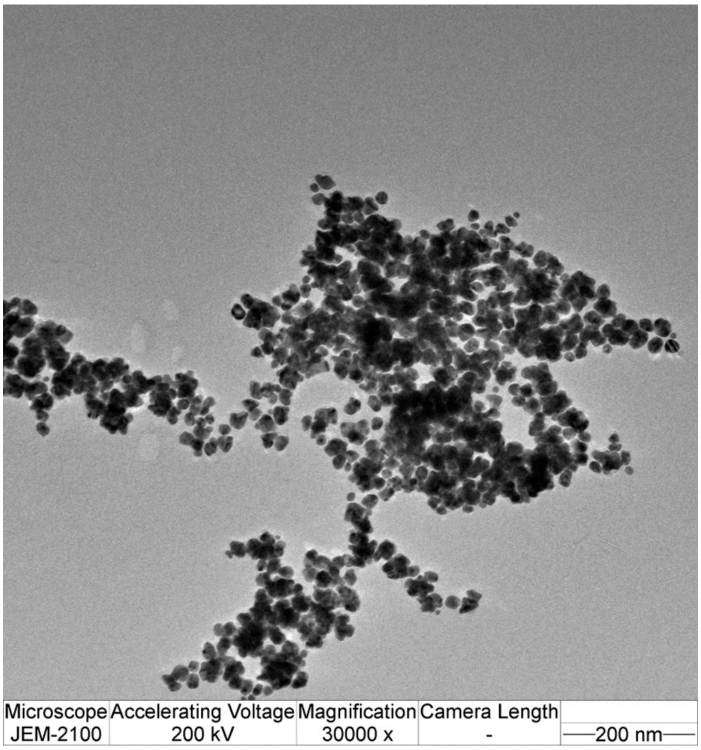

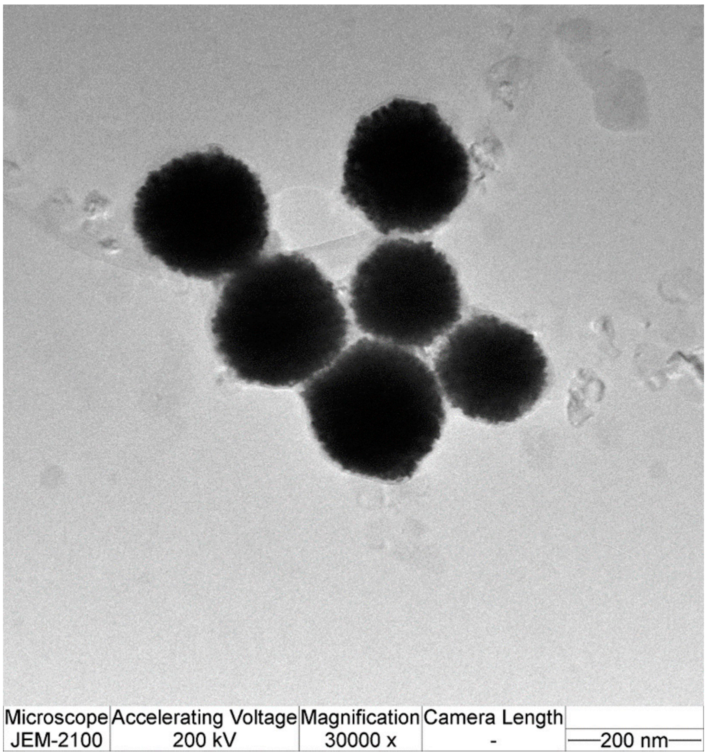

SI Figure S2: The TEM micrographs of AuNPs formed with healthy (top) and cervical cancer affected (bottom) clinical samples.

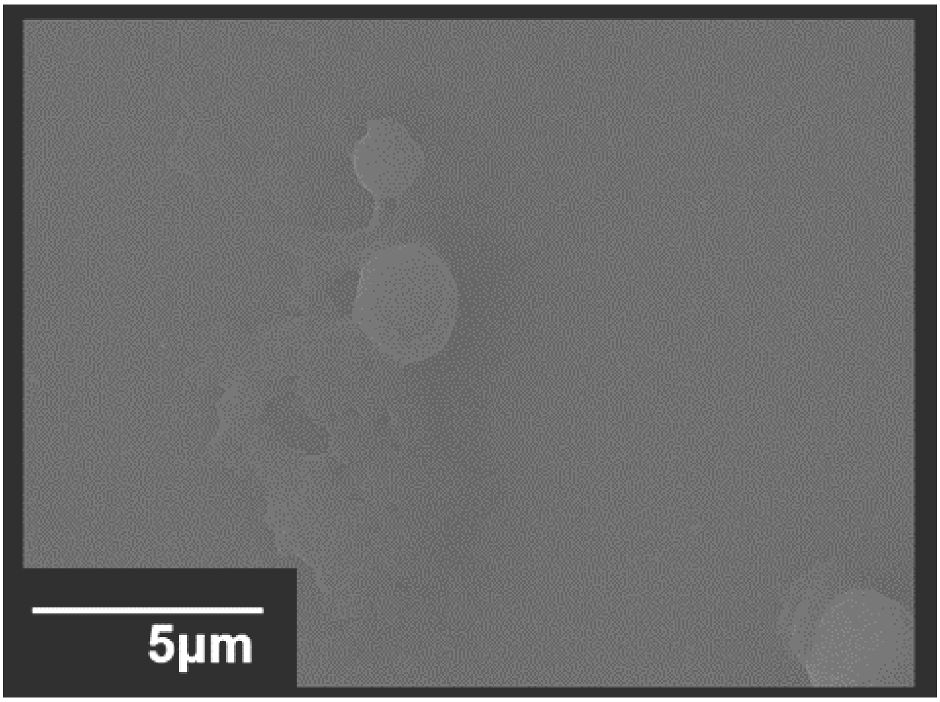

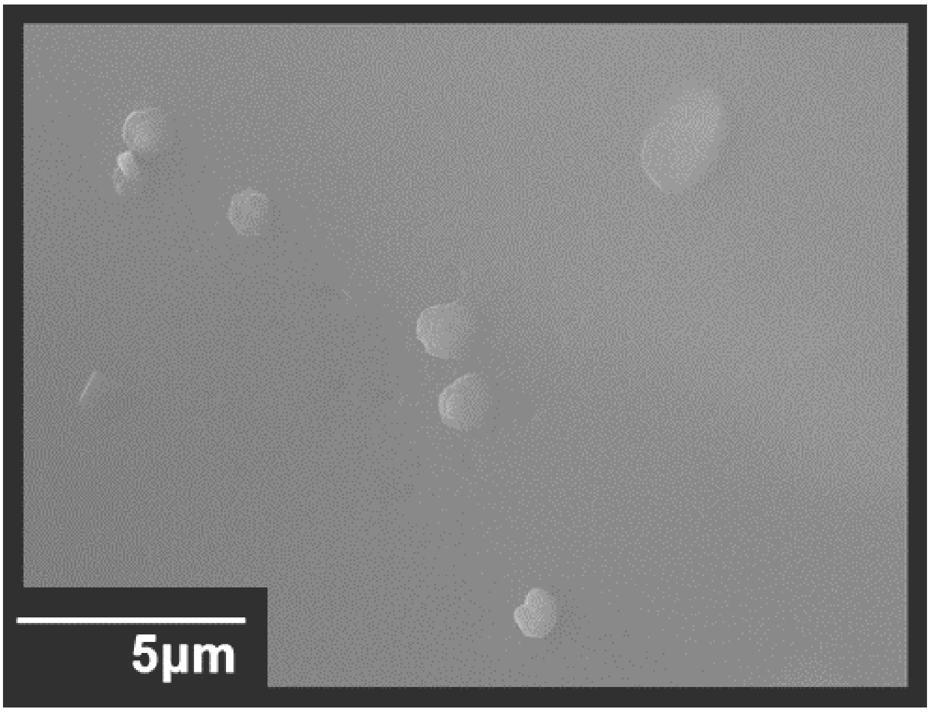

SI Figure S3: The SEM micrographs of healthy (top) and cervical cancer affected (bottom) clinical samples: Cervico-vaginal fluids

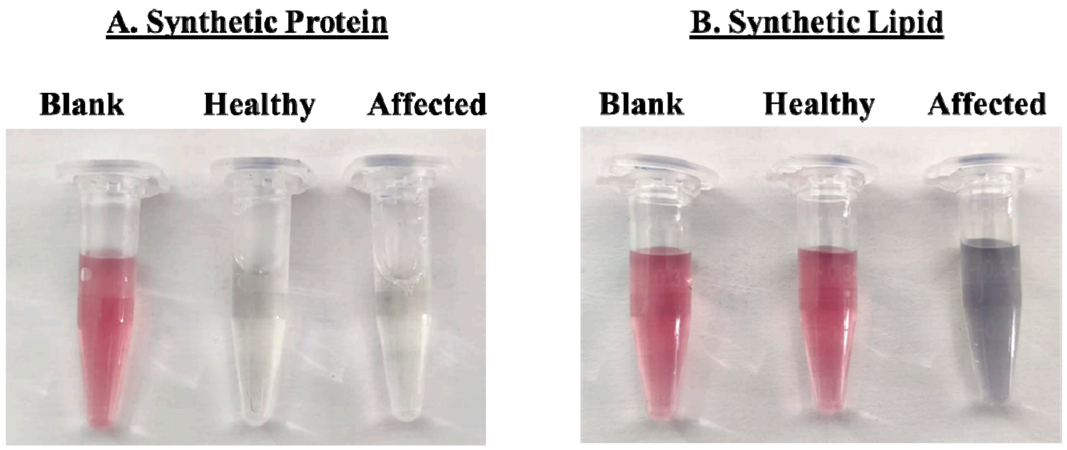

SI Figure S4: The color of AuNPs formed with the A. synthetic protein (albumin) B. synthetic lipid (HSPC) at the average concentrations of protein and lipid found in healthy and affected clinical samples

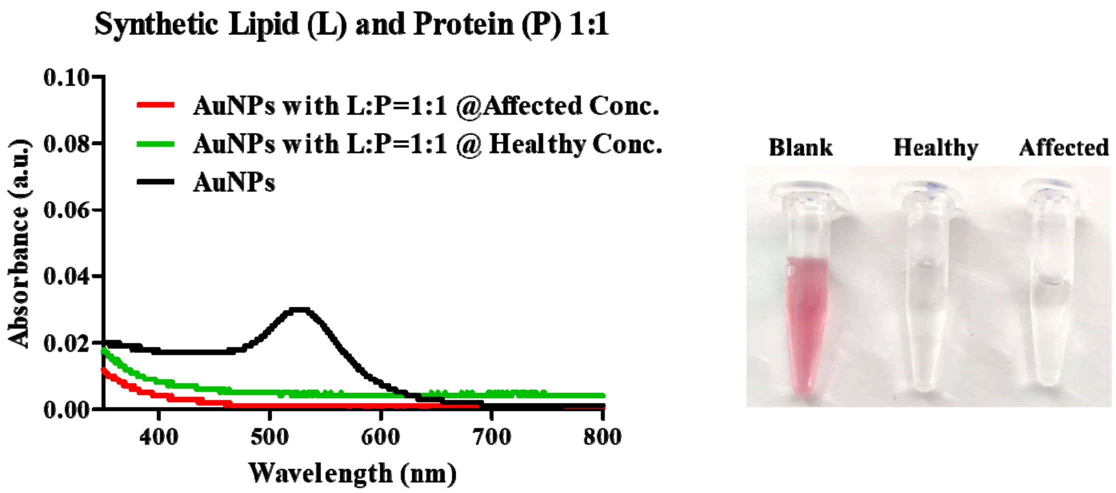

SI Figure S5: The absorbance spectra of AuNPs formed without any lipid (AuNPs) and with lipid and protein in equal concentrations (1:1) found in healthy (AuNPs with L: P=1:1 @Healthy Conc.) and affected (AuNPS with L: P=1:1 @Affected Conc.) clinical samples. The color of AuNPs formed with and without synthetic lipid and protein in the ratio 1:1 are shown in the digital photograph.

SI Table S6: The particle size analysis of the AuNPs formed with synthetic protein (albumin and lipid (HSPC) at average concentrations found in healthy and affected clinical samples.

AuNPs formed with synthetic lipid (HSPC) &/ protein (Albumin)	Average Size (in nm) (Mean ± SEM)
Blank (no lipid or protein)	77.17±13.49
Affected Lipid conc.	350.8±70.88
Healthy Lipid conc.	92.13±22.14
Affected Protein conc.	58.33±10.38
Healthy Protein conc.	245.1±90.71
Affected Lipid and Protein (1:1)	113.2±53.08
Healthy Lipid and Protein (1:1)	92.57±15.02

SI Table S7: The various designs for the self-sampling device and their comparisons.

**Sub Function**	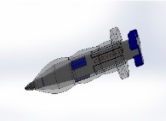	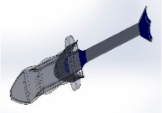	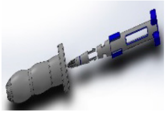	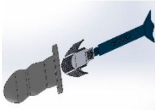	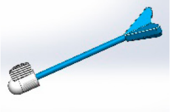
**No of Components**	2	2	3	3	2
**Sample Type**	Semi-solid	Semi-solid	Semi-solid	Semi-solid	Semi-solid
**Rotation**	360^0	$\pm30^0$	360^0	$\pm30^0$	360^0
**Ease of use**	Both hands	Both hands	Both hands	Both hands	Single hand
**Type**	-	-	Detachable bristles	Detachable bristles	Detachable bristles

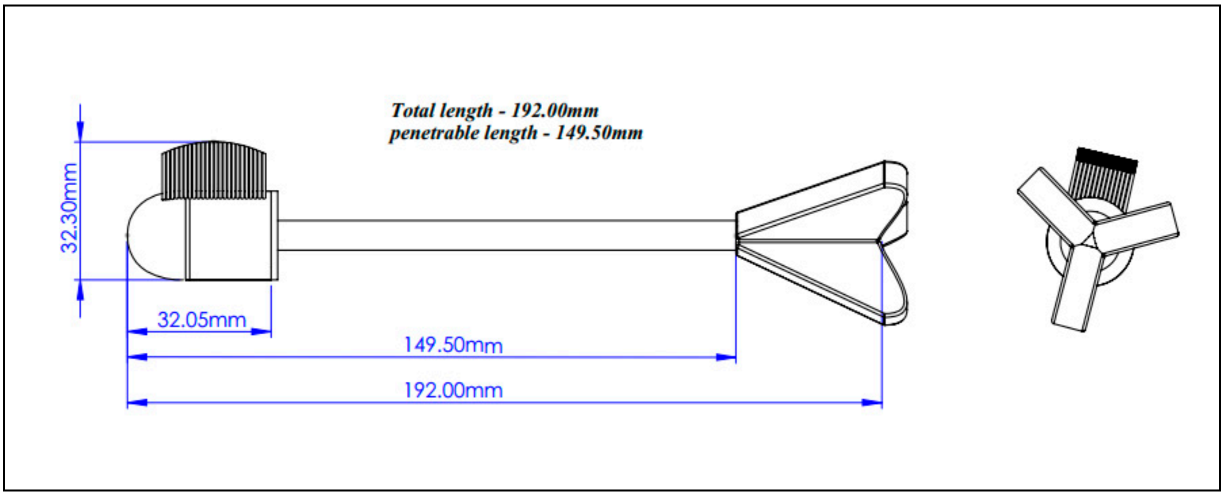

Part	Material	Manufactured by
**Handle**	High density polyethylene (HDPE)	IIT Hyderabad<br>(3D printing laboratory)
**Brush**	Silicone	Quantum labs

SI Figure S6: The detailed dimensions and the materials used for the design 1 of **C**ervi**S**elf.

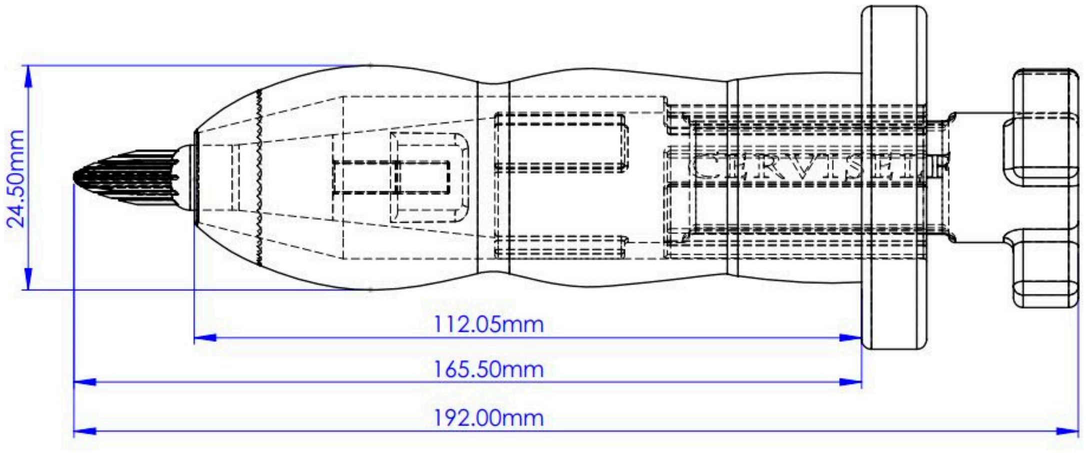

Part	Material	Manufactured by
**Outer casing**	High density polyethylene (HDPE)	IIT Hyderabad<br>(3D printing laboratory)
**Detachable brush**	High density polyethylene (HDPE)	IIT Hyderabad<br>(3D printing laboratory)
**Shaft**	High density polyethylene (HDPE)	IIT Hyderabad<br>(3D printing laboratory)

SI Figure S7: The detailed dimensions and the materials used for the design 2 of **C**ervi**S**elf.

SI Table S8: The specifications of the prototypes and their comparison with existing sample collecting units.

**Specifications**	**Cervi-self (Design 1)**	**Cervix-brush**	**Endo-Cervix Brush**	**Cervi-self (Design 2)**
**Model**	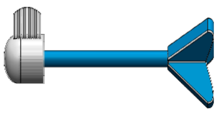	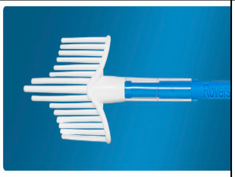	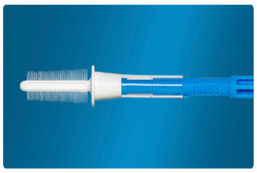	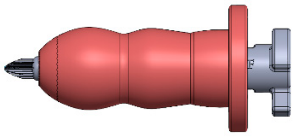
**No. of Components**	2	2	2	3
**Bristle Density**	High	Low	High	High
**Detachable Brush**	Yes	Yes	Yes	Yes
**Examination**	Self	Specialist	Specialist	Self

References:

1. T. Appidi, S. V. Mudigunda, S. Kodandapani and A. K. Rengan, *Nanoscale Advances*, 2020, **2**, 5737-5745.
